# Texture of Hot-Compressed Metastable *β*-Titanium Alloy Ti5321 Studied by Neutron Diffraction

**DOI:** 10.3390/ma17174418

**Published:** 2024-09-07

**Authors:** Bin Gu, Paul Chekhonin, Robert Chulist, Weimin Gan, Werner Skrotzki

**Affiliations:** 1 School of Materials Science and Engineering, University of Science and Technology Beijing, Beijing 100083, China; 2 Institute of Solid State and Materials Physics, Technische Universität Dresden, 01062 Dresden, Germany; 3 Helmholtz-Zentrum Dresden-Rossendorf, 01328 Dresden, Germany; p.chekhonin@hzdr.de; 4 Institute of Metallurgy and Materials Science, Polish Academy of Sciences, 30-059 Krakow, Poland; r.chulist@imim.pl; 5 GEMS at MLZ, Helmholtz-Zentrum Hereon, 85748 Garching, Germany; weimin.gan@hereon.de

**Keywords:** metastable *β*-titanium alloy, hot compression, texture, recrystallization, neutron diffraction

## Abstract

The textures of the *β*- and *α*-phases of the metastable *β*-titanium alloy Ti5321 after hot deformation were investigated by neutron diffraction. A hot-rolled bar was solutionized in the *β*-phase field and then hot compressed above and below the *β*-transus temperature. The initial texture after full recrystallization and grain growth in the *β*-phase field exhibits a weak cube component {001}<100> and minor {112}<110> and {111}<110> components. After hot compression, a <100> fiber texture is observed, increasing in intensity with compression temperature. Below the *β*-transus temperature, dynamic recrystallization of the *β*-phase and dynamic spheroidization of the *α*-phase interact strongly. The texture of the *α*-phase is a <11–20> fiber texture, increasing in intensity with decreasing compression temperature. The mechanisms of texture formation during hot compression are discussed.

## 1. Introduction

Metastable *β*-titanium alloys have gained much research interest due to their unique combination of high strength and good fracture toughness, high strength-to-weight ratio and good fatigue properties [1,2,3,4]. They are usually used in the aerospace industry and other industrial fields [5,6].

The metastable *β*-titanium alloys are very sensitive to processing parameters [7,8,9,10]. Moreover, microstructural heterogeneity and resultant mechanical behavior anisotropy of titanium alloys are generally generated through thermomechanical processing. The thermomechanical processing of metastable *β*-titanium alloys is usually performed near the *β*-transus temperature *T*_β_, where the volume fraction of the *β*-phase (body-centered cubic, bcc) is higher than that of the *α*-phase (hexagonal close-packed, hcp). As a result, the texture evolution and deformation behavior of the *β*-phase has a great influence on the microstructural heterogeneity and resultant mechanical behavior anisotropy of titanium alloys [11,12,13,14]. In addition, the texture (and microstructural heterogeneity), especially after recrystallization, is strongly associated with formability, such as deep drawability, which is very important for industrial production processes [15,16,17,18].

The texture evolution of the *β*-phase in *β*-titanium alloys for different deformation modes has been studied by [11,12,13,14]. After warm rolling to small reductions (≤50%), texture gradients are also small, showing some *α*_bcc_-fiber (crystallographic axis <110> parallel to the rolling direction, RD) and *γ*-fiber (crystallographic axis <111> parallel to the normal direction, ND) texture components [16]. During shape rolling, such as hot bar rolling (92% reduction in cross section of bars), a strong cube component {001}<100> and weak {112}<110> and {111}<110> components formed [11]. For metastable *β*-titanium alloys subjected to hot compression, the texture evolution is related to the Zener–Hollomon parameter Z [17]. Above the *T*_β_, the intensity of the <111> fiber increases with ln Z, whereas that of the <100> fiber slightly decreases.

In studies on recrystallization textures of bcc metals [19,20], it was found that recrystallization can strengthen the intensity of texture components on the *γ*-fiber of interstitial-free (IF) steel after cold rolling. It is commonly believed that the texture components on the *γ*-fiber have higher stored energy, leading to preferable nucleation of recrystallized grains in deformed grains with *γ*-fiber orientations. A high and uniform intensity of the *γ*-fiber is essential for good deep-drawing properties. For Ti–Nb-based *β*-titanium, the {111}<112> component strengthens after solutionizing the alloy in the *β*-phase field for 1 h, and the recrystallizion texture is similar to that of the alloy after warm rolling with 70% thickness reduction [16]. The recrystallization texture of metastable *β*-titanium alloys was also studied by EBSD [11,17,18,21]. After solutionizing in the *β*-phase field for 1 h, a random texture was obtained [11]. After full recrystallization, the texture measured by EBSD may lack accuracy because of low grain statistics. However, there is very limited research on the evolution of the recrystallization textures of metastable *β*-titanium alloys using neutron diffraction. The depth of penetration of neutron radiation can assure the accuracy of the global texture of coarse equiaxed *β*-grains as well as the texture of small volume fractions of second phases. When compression is applied below the *T*_β_, fine equiaxed *α*-phase can precipitate in the metastable *β*-titanium alloys, affecting dynamic recrystallization (DRX) and resultant microtexture of the *β*-phase [22,23]. Generally, referring to DRX in metastable *β*-titanium alloys, discontinuous dynamic recrystallization (DDRX) and continuous dynamic recrystallization (CDRX) are discussed, as well as geometric dynamic recrystallization (GDRX) [24,25]. Also, below the *T*_β_, GDRX grains form, facilitated by the dynamic globularization of *α*-plates, and this is considered as a reason for flow softening of metastable titanium alloys deformed in the (*α* + *β*)-phase field. Consequently, when the volume fraction of the *α*-phase reaches a certain degree (about 50%), the microtexture characteristics in different local areas are not uniform due to the complex DRX mechanism. It is unrealistic to obtain sufficiently large maps by electron backscatter diffraction (EBSD) that include all the texture characteristics in different local areas. As a result, it is very essential to measure the global texture of metastable *β*-titanium alloys deformed below the *T*_β_ through neutron diffraction. In this study, a hot-rolled metastable *β*-titanium alloy Ti5321 with a unique combination of high strength and good fracture toughness was solutionized in the *β*-phase field and then hot compressed above and below the *T*_β_. Our aim was to investigate the global texture formation during DRX of this alloy using neutron diffraction, as well as the effect of the dynamic spheroidization of the *α*-phase on DRX of the *β*-phase.

## 2. Experimental

A forged metastable *β*-titanium alloy Ti5321 (nominal composition Ti-5Al-3V-3Mo-2Cr-2Zr-1Nb-1Fe, wt.%) was produced by the Northwest Institute for Nonferrous Metal Research in China [26]. *T*_β_ of the alloy is (1128 ± 5) K. The forged Ti5321 square bar with a side length of 70 mm was annealed at 1103 K for 30 min. Subsequently, the hot square bar was bar rolled through 10 passes to a round shape with a diameter of 20 mm and air cooled. The reduction in cross section of the hot-rolled (HR) bar was about 92%. Details of the alloy characterization and the bar-rolling process are described in [11].

The HR bar was solution treated (ST) at 1173 K for 1 h followed by water quenching to keep the high temperature microstructure. Subsequently, samples of 8 mm diameter and 12 mm length were hot compressed in a vacuum at temperatures of 1223 K, 1173 K, 1103 K, 1073 K and 1023 K with an initial strain rate of 10^−2^ s^−1^ to a strain of 80% (true strain 1.6) using a Gleeble-3800 thermomechanical simulator (Dynamic Systems Inc., New York, NY, USA). The compression axis (CA) was parallel to the RD of the HR bar. After hot compression, the samples were water quenched. Details about hot compression are provided in [17,18].

The microstructure and microtexture were investigated with a Zeiss ULTRA 55 scanning electron microscope (SEM) (Carl Zeiss, Oberkochen, Germany) using EBSD (HKL Technology, Oxford Instruments, High Wycombe, UK). The EBSD data were analyzed with Channel 5 software. To obtain good sample surfaces for microstructure analysis, the samples were grinded using SiC paper (last step: 4000 grid) in a conventional way followed by electropolishing with an agent consisting of 5% perchloric acid and 95% alcohol. To scan a large area during the EBSD experiments, the step size was set to 3 μm, whereas it was 80 nm for local areas. The operating voltage used for EBSD mapping was 10–20 kV. High-angle and low-angle grain boundaries (HAGBs and LAGBs) of the *β*-phase with misorientations ≥15° and between 3° and 15° were set to black and gray color, respectively. The grain size was determined by the line intercept method of HKL Channel 5 EBSD software. According to Wright et al. [27], recrystallized grains can be determined through the grain orientation spread (GOS) defining the average deviation in orientation between each point in a grain and the average orientation of the grain. A grain was taken as recrystallized for GOS less than 2°.

To determine the phase proportions, high-energy synchrotron diffractograms were measured at the high-energy X-ray beamline HEMS P07B at PETRA III (DESY, Hamburg, Germany) [28] using a PE XRD 1621 detector. The X-ray wavelength was 0.14235 Å, and the beam size was 0.7 × 0.7 mm^2^ [29,30]. Phase proportions were determined from the measured diffractograms with Rietveld refinement using HighScorePlus software and the following crystal structure ICDD data: α—00-001-1197, β—04-004-8475, and α″—01-071-9958. The used parameters along with the so-called countinuous mode ensured good counting statistics with a typical value of goodness of fit parameter lower than 2 for all Rietveld quantifications.

The global textures of the *β*- and *α*-phases were investigated by neutron diffraction. Neutron texture measurements were performed at STRESS-SPEC of MLZ-Heinz Maier-Leibnitz Zentrum, Garching, Germany [31] with a wavelength of 1.717 Å using a Ge (311) single-crystal monochromator. The neutron beam (Ø ≈ 20 mm) penetrated the whole compression sample. Pole figures (PFs) were measured with the STRESS-SPEC robotic system using a continuous scanning routine [32]. The PFs measured were (200), (110) and (211) for the *β*-phase and (0002), (10-10), (10-11) and (11-20) for the *α*-phase. The orientation distribution functions (ODFs) were calculated using the arbitrary defined cells method via LaboTex software (Labosoft). PFs and inverse pole figures (IPFs) were recalculated from the ODFs. Furthermore, LaboTex software was used to export the texture as single orientation files, from which the volume fraction of specific texture fibers were calculated using an in-house written program.

## 3. Results and Discussion

### 3.1. Microstructure and Texture of the β-Phase after Solution Treatment of the Hot-Rolled Bar

**Figure 1:**
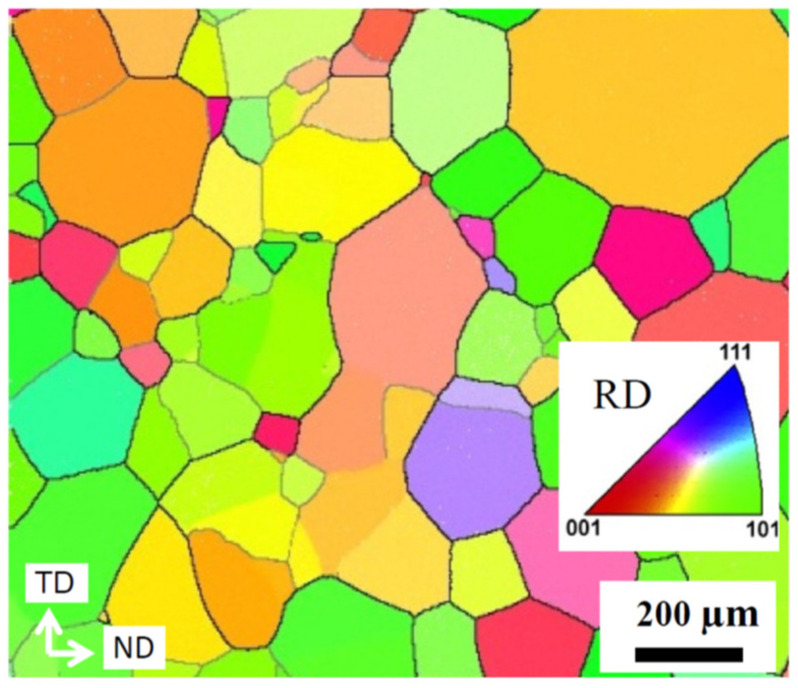
Inverse pole figure map of the ST sample imaged in the RD of the HR bar. (TD = transverse direction, ND = normal direction).

Figure 1 shows the microstructure after solution treatment of the HR bar imaged by EBSD. The exclusively *β*-phase material is fully recrystallized and has a grain size of approximately 165 μm [17].

**Figure 2:**
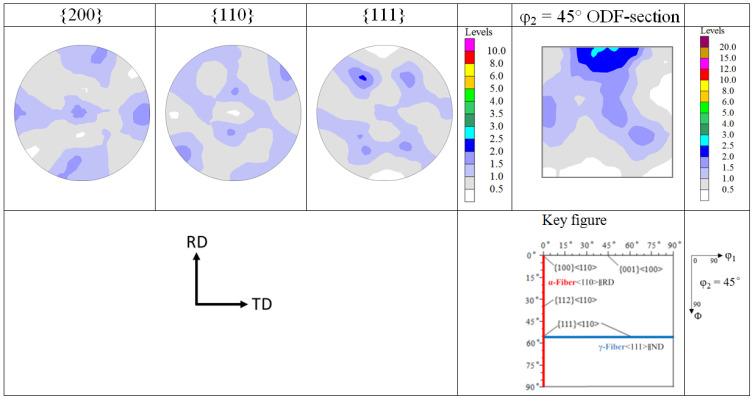
Neutron texture of *β*-phase ST sample represented as PFs and φ_2_ = 45° ODF-section. (RD and TD are directions of the HR sample).

The texture of the *β*-phase ST sample was already analyzed by EBSD on about 450 *β*-grains in [17]. With these rather low grain statistics, the texture was interpreted as almost random. However, with the high penetration depth of neutrons, large samples can be radiated, capturing about more than 10^6^ grains. Figure 2 shows that after complete recrystallization and grain growth during solution treatment of the HR bar, there is still a weak cube component present (volume fraction of about 5%). The cube component appears to be split into two components rotated towards the TD by about 6°. Moreover, there is a very weak coverage of the *α*- and *γ*-fibers with the texture components {112}<110> and {111}<110>, similar to the HR sample [11].

Solution treatment for 1 h in the *β*-phase field leads to recrystallization and grain growth, which is favored by the absence of the *α*-phase. Apparently, this process leads to the weakening and slight splitting of the cube texture. For some other bcc metals, the deformation texture is preserved upon recrystallization, but the intensity of the texture components changes. For example, as in Ti–Nb-based *β*-titanium, after ST in the *β*-phase field, the {111}<112> component on the *γ*-fiber is strengthened, which is similar to the textural characteristics of the alloy observed after rolling to 70% thickness reduction [16]. It is assumed that the {111}<112> nuclei from the dynamic recrystallization process during HR serve as nuclei during static recrystallization. In the present study, the texture components {112}<110> and {111}<110> are inherited from HR and preserved to some extent after static recrystallization, like in other bcc alloys such as Ta, Mo and ferritic steels [15]. However, for the weak cube component {001}<100>, it is assumed that the high ST temperature and long ST time lower the high stored energy of the cube component so that new recrystallization nuclei do not keep the orientation of former cube grains.

### 3.2. Texture of the β-Phase after Hot Compression

**Figure 3:**
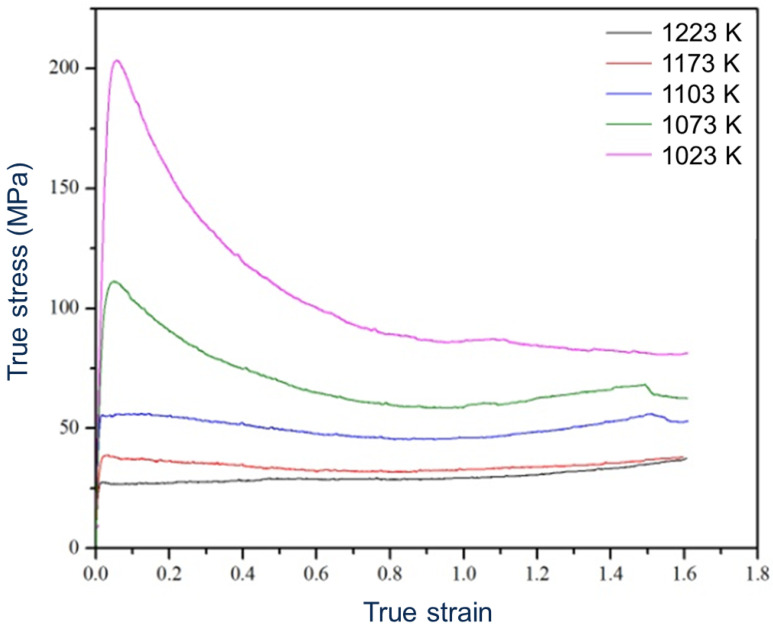
True stress–true strain curves of ST samples hot compressed at temperatures between 1023 K and 1223 K [18].

Figure 3 shows the true stress–true strain curves of ST Ti5321 hot compressed at temperatures between 1023 K and 1223 K. With decreasing deformation temperature, the degree of flow softening increases.

**Figure 4:**
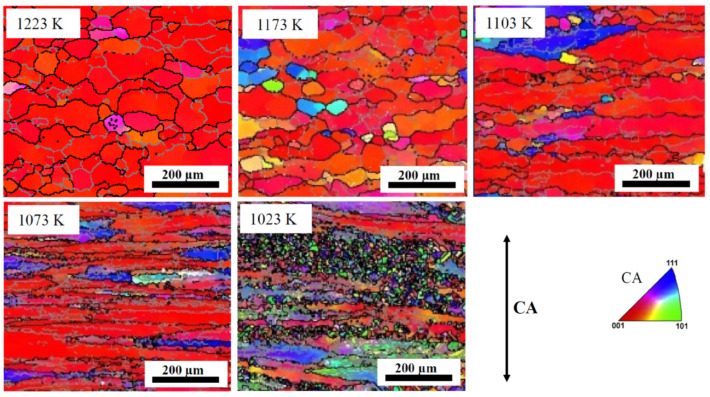
EBSD IPF maps of the microstructure of the *β*-phase of ST samples compressed at different temperatures.

**Figure 5:**
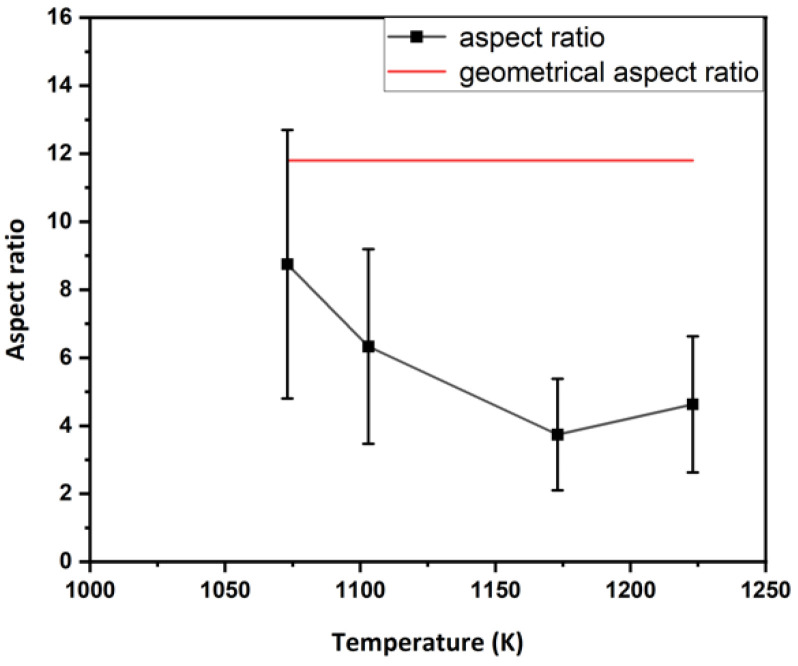
Average *β*-grain aspect ratio as a function of compression temperature. Red line marks the geometric aspect ratio.

**Figure 6:**
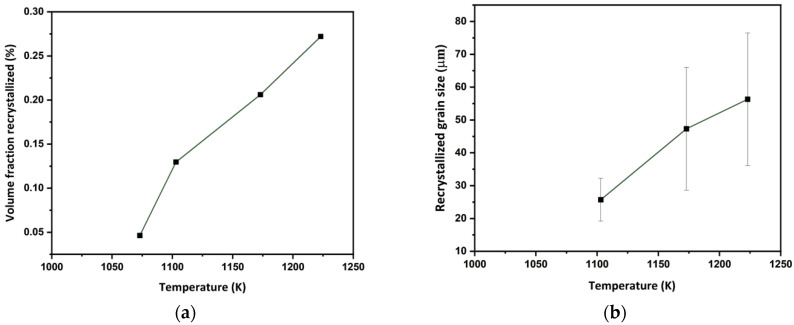
Volume fraction of dynamically recrystallized *β*-grains (**a**) and recrystallized *β*-grain size (**b**) as a function of compression temperature.

Figure 4 shows the microstructure and microtexture of the *β*-phase in ST samples hot compressed at different temperatures. The trends observed with temperature, strain rate and strain have already been extensively discussed in [17,18]. With decreasing temperature, the flattening of the grains perpendicular to the CA increases and, at the lowest temperature, approaches the geometrical aspect ratio corresponding to the compressive strain, assuming a spherical initial grain shape (Figure 5). The change in grain shape is related to DRX. As shown in Figure 6, the volume fraction (Figure 6a) and recrystallized grain size (Figure 6b) increase with compression temperature. While the microstructure can be analyzed easily with EBSD in the *β*-field, it is difficult in the (*α* + *β*-field due to phase transformation. The effect of the *α*-phase on deformation and DRX of the *β*-phase will be discussed below.

**Figure 7:**
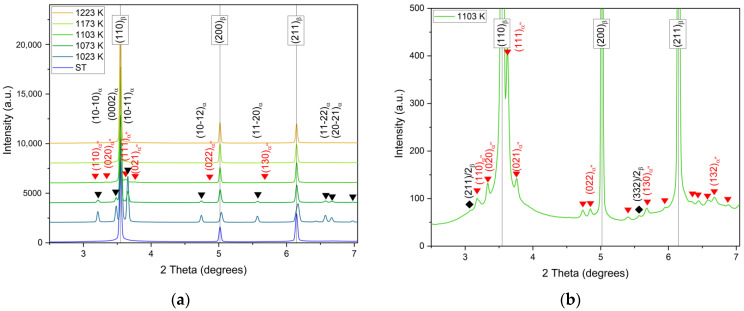
X-ray diffractograms of ST sample and samples compressed at different temperatures (**a**), blow-up of diffractogram for 1103 K (**b**).

According to the diffractograms in Figure 7a, the samples compressed at 1223 K and 1173 K show only bcc *β*-phase (lattice parameter *a* = 3.2513 Å). The sample compressed at 1103 K (25 K below the *T*_β_) shows a martensitic phase transformation to orthorhombic *α*″-phase (volume fraction 8%, lattice parameters *a* = 3.0916 Å, *b* = 4.8923 Å, *c* = 4.7107 Å). A blow-up of the diffractogram for 1103 K is shown in Figure 7b. The samples compressed at 1073 K and 1023 K show only hcp *α*-phase with volume fractions of 12% and 43%, respectively (lattice parameters *a* = 2.9284 Å, *c* = 4.6805 Å, *c/a* = 1.60). The presence of *α*-/*α*″-phase reduces the lattice parameter of the *β*-phase (*a* = 3.2381 Å, clearly seen in Figure 6a for 1023 K, i.e., highest volume fraction of *α*-phase). The texture of the *α*″-phase was not measured. However, if the orientation relationship proposed by Kim et al. [33] and verified by Hayama et al. [34], [100]_α″_||[100]_β_, [010]_α″_||[011]_β_, [001]_α″_||[0-11]_β_ applies, a [100][011> double fiber texture is to be expected, with one fiber dominating in the case of variant selection.

**Figure 8:**
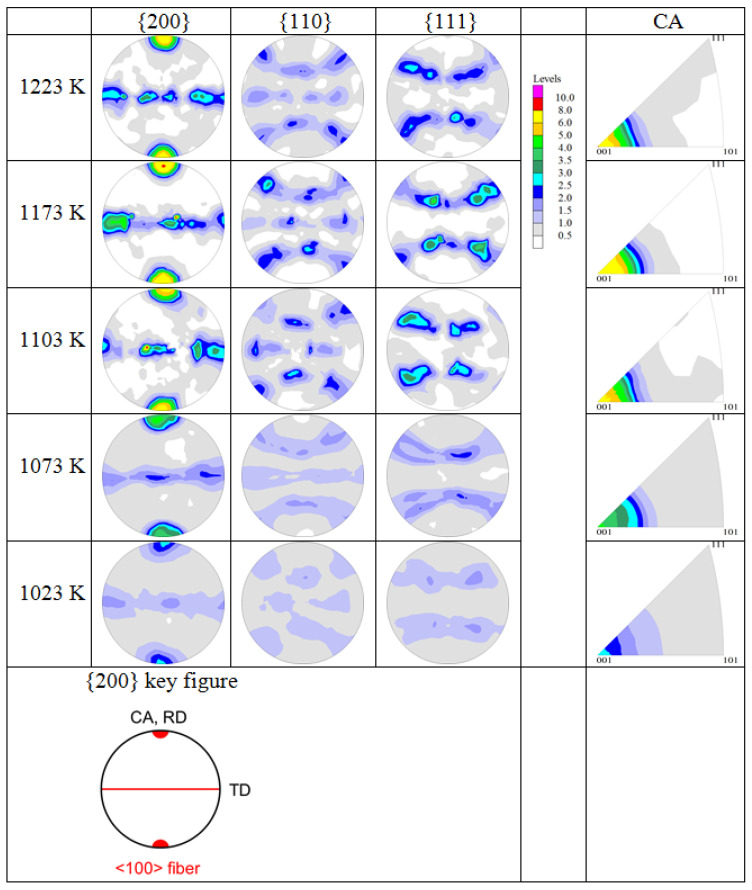
Textures of the *β*-phase after compression at different temperatures represented as PFs and IPFs of CA (CA = compression axis; RD and TD are directions of the HR sample). The intensity levels (in multiples of a random orientation, mrd) are the same for the PFs and IPFs.

**Figure 9:**
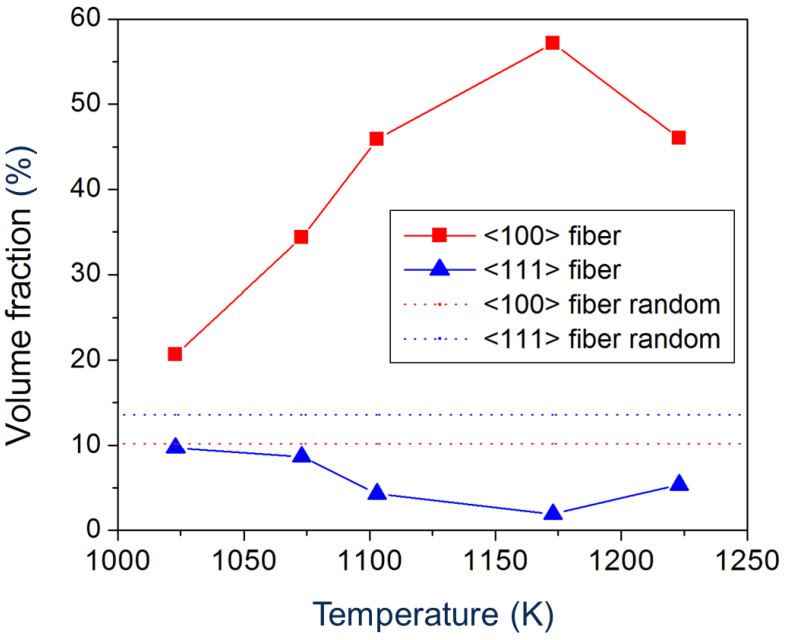
Volume fraction of <100> and <111> texture fibers of *β*-phase as a function of compression temperature. The dotted lines show the volume fraction of these fibers in the presence of a random texture.

The texture characteristics are similar to those found with EBSD for hot-compressed Ti5321 [17] and other metastable *β*-titanium alloys (Ti55511 [21], Ti6246 [35]). Depending on the deformation conditions (temperature, strain rate, strain), at compression temperatures between 1023 K and 1223 K a <100><111> double fiber texture of the *β*-phase forms with <100> dominating (Figure 8). With increasing deformation temperature, the intensity of the <100> fiber increases, whereas that of the <111> fiber decreases (Figure 9). However, under the conditions used in the present study, the volume fraction of the <111> fiber is lower than that calculated for a random orientation distribution. Therefore, only a <100> single fiber exists here. The strengthening of the <100> fiber was attributed to a higher activation of {112}<111> and {123}<111> slip systems [35].

Note that the volume fraction of the <100> fiber measured by neutron diffraction is lower (about 20%) than that measured by EBSD. Neutrons captured the texture of the entire volume of the deformed sample (260 mm^3^), while EBSD only captured a small area (5 mm^2^) in the center of the sample. Assuming that the temperature in the center was higher than at the surface, the volume fraction measured by neutrons should be lower on average.

Comparing Figure 2 and Figure 8, it seems that the <100> fiber of the hot-compressed samples originates from the cube component of the sample after ST. However, its intensity in the hot-compressed samples is much higher than that of the ST sample, except in the sample hot compressed at 1023 K. Therefore, it is assumed that the hot compression strengthens the initial <100> fiber of the recrystallized sample.

### 3.3. Texture of the α-Phase after Hot Compression and Effect of Phase Transformation on Texture Formation in the β-Phase

**Figure 10:**
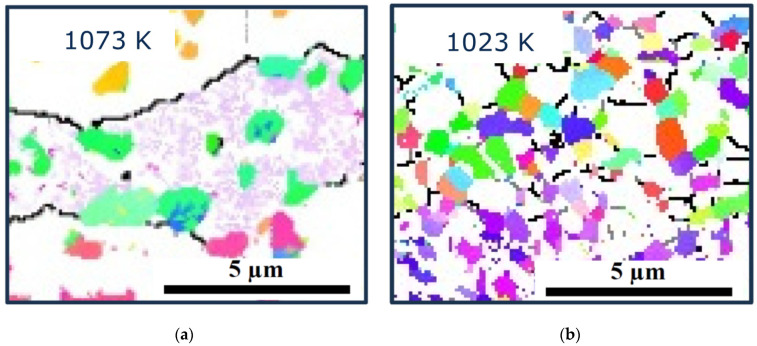
Microstructure of the ST samples hot-compressed at 1073 K (**a**) and 1023 K (**b**). Only the *α*-phase is shown in color.

**Figure 11:**
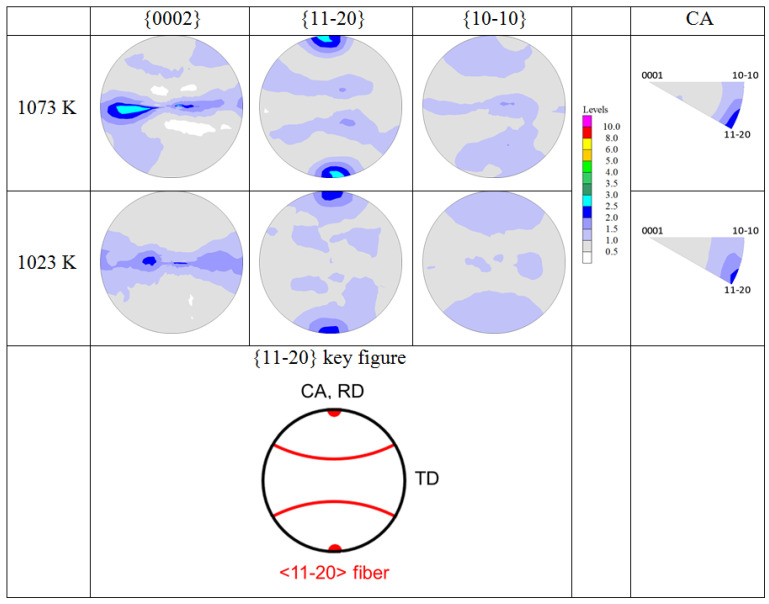
Textures of the *α*-phase in the samples compressed at 1073 K and 1023 K. (CA = compression axis; RD and TD are directions of the HR sample). The intensity levels (in multiples of a random orientation, mrd) are the same for the PFs and IPFs.

Figure 10 shows the microstructures of the samples hot compressed at 1073 K and 1023 K with the *β*-phase removed from the EBSD maps. It can be observed that the almost equiaxed *α*-phase (aspect ratio 1.8 [17]) is mainly spread in the flattened *β*-phase of the sample hot compressed at 1073 K, whereas it is located at the grain boundaries and triple junctions of the fine recrystallized *β*-phase of the sample hot-compressed at 1023 K. The corresponding textures of the *α*-phase measured with neutrons are shown in Figure 11. During hot compression, the *α*-phase develops an obvious fiber texture with <11-20> aligned parallel to the CA (volume fractions: 22% (1073 K), 20% (1023 K)). Comparing Figure 8 with Figure 11, it is obvious that the Burgers orientation relationship (BOR) between the *β*- and *α*-phases ({110}_β_||{0001}_α_ and <111>_β_||<11-20>_α_ does not hold.

During subtransus compression, a <11-20> fiber parallel to the CA was also reported for TIMETAL 834 [36]. Hot compression in the (*α* + *β*)-field close to the *T*_β_ of near *α*-titanium alloy (Ti6242S) for strains up to 70% leads to two texture fibers in the *α*-phase parallel to the CA: strong <11-20> and weaker <20-23> [37]. The <11-20> fiber is mainly due to the activation of prismatic <a> slip, while the <20-23> fiber results from DRX [37]. Based on Schmid factor calculations, Meng et al. [35] attributed the <11-20> fiber to the predominant activation of pyramidal <a> slip. The formation of the <11-20> fiber in the present alloy could have been caused accordingly, and thus destroyed the BOR [38].

The formation of *α*″-phase of the sample compressed at 1103 K might βhave occurred during quenching after deformation in the *β*-field (just above the *T*_β_) due to probable adiabatic heating. This may be concluded from the fact that the volume fraction of the <100> fiber is as high as after deformation in the *β*-field. Moreover, the flow curve resembles those above the *T*_β_ (Figure 3). The formation of *α*″-phase was reported due to water quenching of Ti-35Nb alloys from 1273 K (above the *T*_β_) [34] and helium gas quenching of Ti6246 [35]. The fact that no *α*″-phase is observed in the ST sample might indicate that deformation is important to promote heterogeneous nucleation.

**Figure 12:**
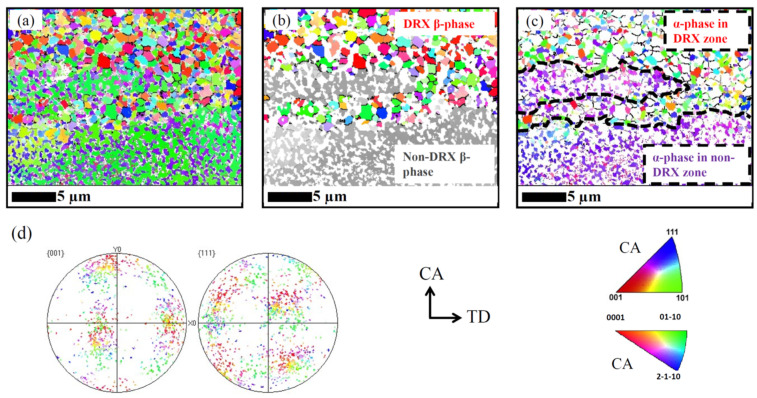
EBSD maps of the samples hot compressed at 1023 K: (**a**) *α*- and *β*-phases are shown in color; (**b**) only the DRX *β*-phase is shown in color; (**c**) only the *α*-phase is shown in color; (**d**) PFs of the DRX *β*-phase in (**b**).

Figure 12 shows an IPF map of the sample hot compressed at 1023 K (105 K below the T_β_) in the two-phase region. In Figure 12a, the *α*- and *β*-phases are shown together, while in (b) and (c) they are separately imaged. Moreover, DRX and non-DRX zones are marked. Figure 12d shows the PFs of the DRX *β*-phase. The microtexture of the *β*-phase is similar to the global texture shown in Figure 8, but slightly rotated around the CA.

With decreasing compression temperature, the volume fraction of the <100> fiber decreases. At 1023 K, the intensity of <100> fiber is extremely low. As discussed above, the DRX mechanism of *β*-grains is strongly affected by the *α*-phase when the volume fraction of the *α*-phase reaches a certain level. According to Rietveld analysis of the diffractogram in Figure 7a, the volume fractions of the *α*-phase are 12% and 43% at 1073 K and 1023 K, respectively. These values have to be compared with the area fractions determined by EBSD in [17]: 21% and 35%. Evidently, EBSD captures the inhomogeneities of the microstructure.

From the two different changing trends of the true stress–true strain curves, it can be concluded that there are different deformation mechanisms when samples are compressed at different temperatures (Figure 3). The flow curves above the *T*_β_ quickly rise to a plateau followed by steady-state flow. Sakai et al., 2014 [23] revealed that this deformation behavior is dominated by dynamic recovery (DRV), which is the main restoration mechanism in *β*-Ti alloy [39]. In this study, before a strain of about 0.02, the flow stress increase is due to strain hardening that is associated with the generation, movement and multiplication of dislocations [40]. With increasing strain, the rate of DRV increases. Above a strain of 0.02, strain hardening is balanced by DRV, which includes the annihilation and rearrangement of dislocations, leading to the formation of a subgrain structure. Now, dynamic equilibrium is reached, shown as steady-state flow. Generally, steady-state flow is found in hot deformation at relatively low strain rates.

A relatively low degree of flow softening (about 30% stress drop) is found in Ti55531 [41] and Ti7333 [42] deformed at higher strain rates (>10^−1^ s^−1^). In this study, the relatively low strain rate (10^−2^ s^−1^) avoids flow instability to some degree due to a relatively high thermal conductivity. As a result, there must be other reasons for the high degree of flow softening (about 44% and 54% stress drop at 1073 K and 1023 K, respectively) occurring during hot compression below the *T*_β_ in Ti5321.

In Ti1023, the flow softening similar to Ti5321 is considered to be due to the break-up of Widmanstätten platelets during isothermal deformation [24]. In light of this, the flow softening observed in this study can be explained as follows. Before compression, lamellar *α*-grains formed during the 2 min heat treatment. In the initial stage of hot compression, peak hardening is caused by dislocations piling up at *α/β* interphase boundaries. According to the study of Li et al. [43], the lamellar *α*-grains become kinked and at a critical stress become fragmented via breakthrough of *β*-phase at *α*-grain boundaries. After additional hot compression, the divided prior *α*-grains become more equiaxed by diffusion (Figure 10 and Figure 12). Thus, the dynamic globularization process based on stress-induced diffusion contributes much to flow softening.

Moreover, Chen et al. [13] mentioned that during further hot compression, dislocations pinned at *α/β* interphase boundaries locally increase the dislocation density, which promotes DRX. This was experimentally confirmed in this study, as shown in Figure 12. At 1023 K, very fine DRX *β*-grains (~0.5 μm) form at *β*-grain boundaries and/or triple junctions (Figure 10b) at 1023 K. Warchomicka et al. [41] recognized this as GDRX. The new DRX grains were considered to result in significant flow softening.

## 4. Conclusions

The texture of metastable *β*-titanium alloy Ti5321 during static recrystallization and grain growth as well as hot-compression was investigated by neutron diffraction, which enabled texture measurements of statistical relevance of coarse-grained materials with low-volume fractions of fine phases. The main conclusions from this study are as follows:

1. After solution treatment of the hot-rolled alloy, in the *β*-phase there is still a weak cube component present as well as weaker *α*_bcc_-fiber components. The intensity of the cube component is much lower than that of the hot-rolled bar.
2. Under the deformation conditions used during hot compression, a <100> fiber texture of the *β*-phase forms. With increasing deformation temperature, the intensity of the <100> fiber increases. The formation of a weak <100> fiber of the *β*-phase is associated with a certain level of *α*-participation.
3. After hot compression, the texture of the *α*-phase shows an obvious fiber texture with <11-20> aligned parallel to the CA. The Burgers orientation relationship between the *β*- and *α*-phases is not maintained. Instead of the hcp *α*-phase, the orthorhombic martensitic *α*″-phase was observed in the sample, which was compressed near the *β*-transus temperature (probably just above it because of adiabatic heating). It is assumed that this phase nucleated heterogeneously during quenching from the deformed *β*-phase.
4. The strong flow softening is due to both the dynamic globularization process based on stress-induced diffusion and the formation of fine GDRX *β*-grains.

## Data Availability

Data are contained within the article.
